# Variability in the Clinical Effects of the Omega-3 Polyunsaturated Fatty Acids DHA and EPA in Cardiovascular Disease—Possible Causes and Future Considerations

**DOI:** 10.3390/nu15224830

**Published:** 2023-11-18

**Authors:** Charalambos Michaeloudes, Stephanos Christodoulides, Panayiota Christodoulou, Theodora-Christina Kyriakou, Ioannis Patrikios, Anastasis Stephanou

**Affiliations:** School of Medicine, European University Cyprus, Nicosia 2404, Cyprus; s.christodoulides@external.euc.ac.cy (S.C.); pa.christodoulou@euc.ac.cy (P.C.); t.kyriakou@euc.ac.cy (T.-C.K.); i.patrikios@euc.ac.cy (I.P.); a.stephanou@euc.ac.cy (A.S.)

**Keywords:** *n*-3 polyunsaturated fatty acids, atherosclerosis, molecular mechanisms, epigenetics, personalized medicine

## Abstract

Cardiovascular disease (CVD) that includes myocardial infarction and stroke, is the leading cause of mortality worldwide. Atherosclerosis, the primary underlying cause of CVD, can be controlled by pharmacological and dietary interventions, including *n*-3 polyunsaturated fatty acid (PUFA) supplementation. *n*-3 PUFA supplementation, primarily consisting of eicosapentaenoic acid (EPA) and docosahexaenoic acid (DHA), has shown promise in reducing atherosclerosis by modulating risk factors, including triglyceride levels and vascular inflammation. *n*-3 PUFAs act by replacing pro-inflammatory fatty acid types in cell membranes and plasma lipids, by regulating transcription factor activity, and by inducing epigenetic changes. EPA and DHA regulate cellular function through shared and differential molecular mechanisms. Large clinical studies on *n*-3 PUFAs have reported conflicting findings, causing confusion among the public and health professionals. In this review, we discuss important factors leading to these inconsistencies, in the context of atherosclerosis, including clinical study design and the differential effects of EPA and DHA on cell function. We propose steps to improve clinical and basic experimental study design in order to improve supplement composition optimization. Finally, we propose that understanding the factors underlying the poor response to *n*-3 PUFAs, and the development of molecular biomarkers for predicting response may help towards a more personalized treatment.

## 1. Introduction

### 1.1. Cardiovascular Disease

Cardiovascular disease (CVD), including myocardial infarction and ischemic stroke, is the leading cause of death worldwide. Atherosclerosis, which entails the formation of an atheromatic plague due to build-up of lipids and fibrous tissue in the large arteries, is the primary underlying cause of CVD [1]. Stenosis caused by the atheromatic plague, as well as thrombosis due to plague rupture, can restrict blood flow in major vessels leading to myocardial infarction or ischemic stroke [2].

Myocardial infarction, caused by myocardial ischemia, involves myocardial necrosis, as well as other cell death-activating pathways due to oxygen deprivation [3]. The main pathological cause of myocardial infarctions is associated with coronary artery disease, which involves coronary artery blockage due to accumulation of atherosclerotic plagues [4]. Myocardial damage can eventually lead to heart failure, which involves anatomical and functional myocardial abnormalities that limit ventricular filling or blood ejection, and results in failure to satisfy the underlying needs of circulation [5]. Ischemic stroke involves a reduction in the blood flow to part or all of the brain, leading to tissue damage and some degree of neurological damage. The major cause of ischemic stroke is the rupture of atherosclerotic plagues and the formation of thrombosis in the carotid artery [6].

### 1.2. Atherosclerosis

Atherosclerosis is primarily a lipid-driven process caused by accumulation of low-density lipoprotein (LDL) and remnant lipoprotein particles in focal areas of arteries, and an associated inflammatory process particularly at regions of disturbed non-laminar flow at artery branch-points. The defined most frequent risk factors are: high LDL cholesterol (LDL-C), hypertension, diabetes mellitus, smoking, age, and family history. Sedentary lifestyle, obesity, diets high in saturated and trans fats, and specific genetic abnormalities may also increase the risk [7,8,9].

Atherosclerosis primarily develops due to trapping of lipids in the intima by the extracellular matrix, causing a modification that drives chronic inflammation at vulnerable sites in the arteries. This process, which is involved in all stages of atherogenic progression, starts with nascent fatty streaks in the artery intima, which develop into fibrous plaques and then into rupture-prone atherosclerotic lesions [10,11]. At the molecular level, LDL in the intima becomes oxidized and drives pro-inflammatory processes that attract monocytes to the artery wall, where they differentiate into macrophages. Macrophages take-up lipoproteins using LDL scavenging receptors, forming foam cells that create the atherosclerotic lesion. Inflammatory mediators also recruit vascular smooth muscle cells, which produce extracellular matrix proteins creating a fibrous cap that overlies the lesion, forming an atherosclerotic plague [12]. Reduced synthesis of extracellular matrix proteins by smooth muscle cells, and increased production of matrix metalloproteases by foam cells may lead to fibrous cap thinning and plague rupture leading to thrombosis [13,14,15]. In advanced plagues, there is increased smooth muscle cell and macrophage cell death, through apoptosis or necroptosis [16,17]. Failure of macrophages to clear apoptotic and necrotic cells by efferocytosis, leads to the formation of the necrotic core that precipitates inflammation and fibrous cap thinning [12,18].

Treatment for coronary atherosclerosis involves measures to encourage regression and stop the growth and rupture of atherosclerotic plaques. Treating risk factors such as elevated LDL-C, high blood pressure, and diabetes, through diet, exercise, smoking cessation, and pharmacological management are the key strategies for controlling atherosclerosis [19,20,21]. Statins are the mainstay for lowering LDL-C and preventing cardiovascular events and mortality. Angiotensin-converting enzyme inhibitors, angiotensin II receptor blockers, diuretics, beta-blockers, calcium channel blockers, and vasodilators are also used for blood pressure management [22,23,24].

### 1.3. Dietary Management of Atherosclerosis

Atherosclerosis can be controlled by a healthy diet that is rich in fibre, monounsaturated fats, oily fish, fruits, and vegetables, and low in saturated and trans fats [25]. *n*-3 polyunsaturated fatty acids (PUFAs) have received considerable attention for their potential in modulating key risk factors of CVD, including triglyceride levels, lipoprotein oxidation, vascular inflammation and thrombogenesis. Nonetheless, clinical studies investigating the effects of *n*-3-PUFAs on cardiovascular health show conflicting findings [26]. A better understanding of the factors underlying the variability in the effects of *n*-3-PUFAs in clinical studies will allow us to tailor supplementation regimes in order to achieve greater benefit for the patients. In this review, we will discuss possible causes of the reported variability, by focusing on atherosclerosis. Furthermore, we provide suggestions for improving pre-clinical and clinical studies in order to better understand and optimize *n*-3 PUFA supplementation for preventing CVD.

## 2. Current Status of Knowledge

### 2.1. Biochemistry and Structural Morphology of *n*-3 PUFAs

*n*-3 (omega-3) PUFAs are a family of long chain cis polyunsaturated fatty acids [27]. The term *n*-3 reflects the fact that the first double bond is situated three carbon atoms from the methyl terminal group [27]. Alpha linolenic acid (ALA; C18:3) is precursor to the longer chain (LC) *n*-3 PUFAs, eicosapentaenoic acid (EPA; C20:5) and docosahexaenoic acid (DHA; C22:6) [28]. ALA is ‘essential’ and must be included in our diet since it cannot be synthesised de novo in the body. Oily fish are the main source of LC *n*-3 PUFAs [29]. *n*-6 (omega-6) FAs are the second known important family of PUFAs. Linoleic acid (LA; C18:2) is a precursor to the LC *n*-6 PUFA arachidonic acid (C20:4). The main sources of *n*-6 PUFAs include the common vegetable oils used in cooking, such as sunflower and soybean oil, as well as foods derived from livestock animals and poultry [30].

It has been widely accepted that the present Western diet is ‘deficient’ in *n*-3 PUFAs with a ratio of *n*-6 to *n*-3 of 15-20:1, which is far higher than the optimal 4:1 ratio and the ideal 1:1 ratio [31]. However, reducing *n*-6 PUFA intake is not a prerequisite to achieve the optimum ratio. According to Zhao et al., *n*-6 PUFA intake can be increased without inducing any adverse effect provided that adequate *n*-3 PUFAs are consumed. Moreover, sufficient consumption of *n*-6 PUFAs is important for reducing LDL-C concentrations and therefore, a sufficient intake of both *n*-3 and *n*-6 PUFAs is crucial for CVD risk reduction [32].

### 2.2. Clinical Evidence on the Effects of *n*-3 PUFAs on Different CVDs

**Figure 1:**
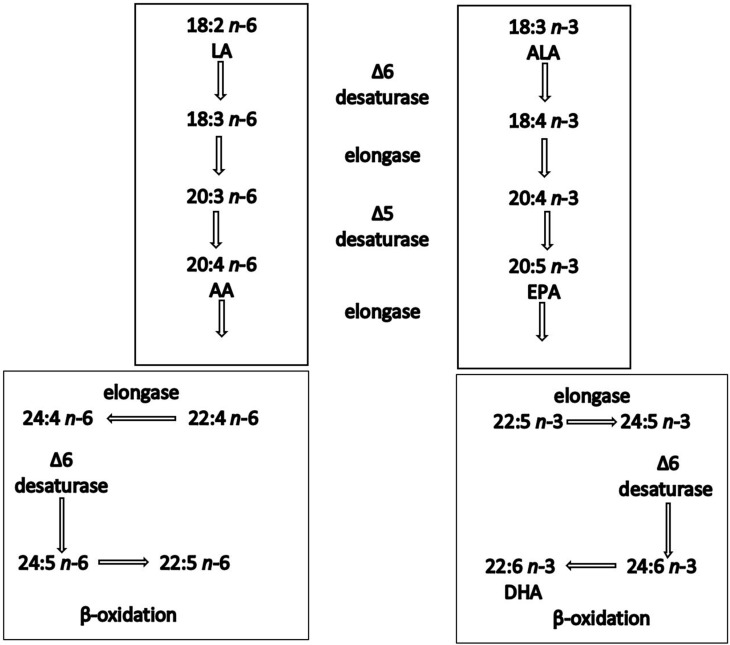
Synthesis of *n*-6 and *n*-3 polyunsaturated fatty acids in humans. Both linoleic acid (LA) and a-linolenic acid (ALA) are elongated, desaturated and β-oxidised using the same enzyme system.

Epidemiological studies have shown a positive correlation between oily fish intake and beneficial cardiovascular effects [33]. The evidence of the benefits of oily fish is stronger in secondary than in primary prevention settings [34]. For instance, GISSI Prevenzione investigators (1999) showed that dietary supplementation with LC *n*-3 PUFAs (1 g/day) in patients after myocardial infarction reduced total mortality by 21% and sudden death by 45%. In addition, a recent pooled analysis of four cohort studies showed an association between oily fish intake and lower risk of mortality among patients with prior CVD [35]. These benefits have been attributed to the LC *n*-3 PUFAs, EPA and DHA, primarily found in oily fish [36]. ALA is generally far less effective at inducing biological effects, partly because of its inefficient conversion (<5%) to EPA and DHA in humans [28]. ALA is inefficiently converted into the LC *n*-3 PUFAs partly because of the large and increasing amounts of *n*-6 fatty acids present in our diet, which compete for the same enzymes, shown in the metabolic pathway [37,38] (Figure 1).

The beneficial effect of the LC *n*-3 PUFAs on reducing the risk of cardiac mortality appears to be due to their incorporation into cardiomyocyte phospholipids at the expense of arachidonic acid [39,40]. The presence of *n*-3 PUFAs, particularly EPA, in the cell membranes can lead to the generation of different eicosanoids which can be more cardioprotective than those resulting from the arachidonic acid cascade [41,42,43]. Several potential mechanisms for the cardioprotective effect of omega-3 fatty acids have been proposed with the effect on blood lipids, such as triglycerides (TG), LDL, and HDL, associated with risk of atherosclerosis, being the most scientifically proven [44,45]. Particularly, the most consistent effect of *n*-3 PUFAs is the reduction in serum TG [46].

There is a substantial confusion among the public and health professionals regarding the overall effects of *n*-3 PUFAs on blood lipids [47]. It has been suggested that elevated TG levels are independent risk factors for the progression of CVD [48]. In addition, smaller diameter LDL and HDL particles have been associated with increased risk [49,50]. The ‘Omega-3 index’ [51], which describes the content of EPA and DHA in red blood cell (RBC) membranes, expressed as a proportion of total FAs, has been considered a risk factor for death from coronary heart disease (CHD) [52].

#### 2.2.1. Effect on Triglycerides

According to Milte et al., circulating TG levels constitute an independent risk factor for CVD and are correlated with the severity and development of atherosclerosis [53]. However, Torrejon et al., argued that unlike the well-established role of LDL-C in the development of CVD, the role of circulating TG concentrations in CVD development remains controversial [54]. Nevertheless, according to the British Nutrition Foundation (BNF) (2005) [55], an indirect effect of TGs on CVD risk seems to be the case. Research has shown that a reduction in TG levels leads to a lower abundance of small, dense LDL-C and therefore a reduced CVD risk (BNF, 2005) [55].

The most consistent effect of *n*-3 PUFAs is the decrease in fasting and postprandial serum TGs [48,54]. However, the exact dose as well as exact duration of intervention, which can give the optimum TG-lowering effect is still unclear [53]. So far, the majority of the studies have demonstrated a significant reduction in TGs (25–30%) following treatment with doses ≥ 3 g/day of LC *n*-3 PUFAs (EPA and DHA) mainly in the form of fish oil [56]. In contrast, there is evidence showing a much lower effect following treatment with lower doses [57]. The duration of studies ranged between 6 and 104 weeks, and the optimal duration of intervention remains unclear since no difference was found in TG levels associated with longer trial duration (>16 weeks) compared to shorter trial duration (≤16 weeks) [33,58].

A double-blind randomized placebo controlled parallel study showed that DHA supplementation (3 g/day) for 45 days significantly decreased fasting TG levels of hypertriglyceridaemic men by 25–30%. Conversely, a double-blind placebo-controlled study [57] using a LC *n*-3 PUFA dose (1.8 g EPA and 0.3 g DHA) per day, which approximated the daily dietary intake upper limit (2 g of LC *n*-3 PUFAs) of the current UK guideline range (Scientific Advisory Committee on Nutrition, 2004) [59], had no effect on TG levels of normotriglyceridaemic individuals. Yusof et al. suggested that apart from the lower dose, the fact that the oil used was relatively poor in DHA might have contributed to the lack of effect. It is now believed that DHA is more potent in lowering TGs than EPA [60]. Furthermore, it has been shown that the DHA TG-lowering effect is greater in hypertriglyceridaemic individuals compared with normotriglyceridaemic individuals [61,62]. A comprehensive meta-analysis (16511 participants in 47 studies included) assessing the role of *n*-3 PUFAs in treating hyperlipidaemia, demonstrated an average reduction of 14% in TG levels of hypertriglyceridaemic individuals following 6-month treatment with an average daily intake of 3.25 g of EPA and/or DHA [33]. However, this dose exceeds the safety limit (3 g/day) approved by the US food and drug administration (FDA) [63].

#### 2.2.2. Effect on Low-Density and High-Density Lipoprotein Particle Size

Smaller diameter and denser LDL particles, such as LDL-3 subfraction, have been shown to be more susceptible to oxidation [50]. In addition, they have an increased ability to penetrate the intima than larger, less dense LDL particles, such as LDL-1 and LDL-2 subfractions [50] and have, therefore, been associated with an increased coronary heart disease risk [50]. A very recent systematic review concluded that small dense LDL are associated with an increased CVD risk [64]. According to Torrejon et al., particle size is significantly increased with DHA supplementation. DHA supplementation reduces plasma TGs, contributing to a reduction in the number of small, dense LDL particles and subsequently to decreased CVD risk [49].

Larger, more cholesterol-rich HDL particles (e.g., HDL-2 subfraction) are thought to facilitate a more efficient reverse cholesterol transport compared with the smaller, less buoyant subfraction HDL-3, making them more cardioprotective [50,65,66]. The HDL-2 subfraction has been shown to be increased in response to DHA, whilst it is not affected by EPA [54,65]. This was confirmed by a recent systematic review [67].

#### 2.2.3. Effect on ‘*n*-3 Index’

**Figure 2:**
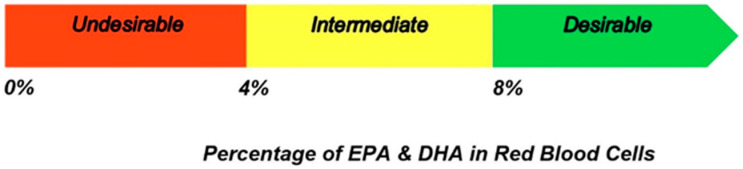
Proposed risk zones for the ‘omega-3 index’.

Harris and von Schacky (2004) were the first to propose that the content of EPA and DHA in RBC membranes, expressed as a proportion of total FAs, termed the ‘omega-3 index’, can be considered as a risk factor for coronary heart disease and sudden cardiac death mortality [51]. Specifically, an ‘omega-3 Index’ level of ≥8% is a reasonable preliminary target value for reducing coronary heart disease risk. On the other hand, an ‘omega-3 Index’ < 4% has been implicated with a 10-fold greater risk of sudden cardiac death compared with an ‘omega-3 Index’ of ≥8% (Figure 2) [68,69].

An increased proportion of the LC *n*-3 PUFAs (EPA and DHA) in erythrocyte membranes following consumption of oily fish has been demonstrated [70]. A randomised, double-blind, placebo-controlled parallel trial investigated the association between increases in erythrocyte DHA content and changes in blood lipids in 67 healthy individuals following DHA intake for 3 months [53]. Participants were randomised to receive 0.52 g, 1.04 g, 1.56 g DHA or 1 g Sunola oil (control). At the end of the 3-month intervention the 0.52 g, 1.04 g, 1.56 g DHA doses increased the proportion of DHA in erythrocytes to a dose-dependent manner (7.1%, 7.9% and 9% of total FAs, respectively) [53].

## 3. Factors Implicated in the Efficacy of PUFAs

### 3.1. Variability in the Clinical Effects of *n*-3 PUFAs

Following supplement uptake, *n*-3 PUFAs are incorporated into cell membranes and plasma lipids in a dose- and time-dependent manner, replacing other fatty acid types, including *n*-6 PUFAs [71]. *n*-3 PUFA incorporation lead to changes in cell signaling by affecting the cell membrane structure and fluidity and changing the function of cell surface receptors and ion channels. The effects of *n*-3 PUFAs on atherosclerosis are conferred through molecular mechanisms that involve changes in the cell membrane composition, eicosanoid synthesis, transcription factor activity and gene expression [72]. EPA and DHA are known to have differential metabolism, and divergent effects on the molecular mechanisms of atherosclerosis. Antagonism between the two types of *n*-3 PUFAs, may explain the conflicting findings of large clinical studies on the effects of *n*-3 PUFAs on CVD risk factors [73,74,75,76]. The effects of *n*-3 PUFAs on atherosclerosis risk factors depend on different considerations including their dose and length of supplementation, EPA/DHA composition and formulation [77]. Understanding how these factors affect their incorporation into the lipid pool, their metabolism and their downstream molecular effects will allow more effective design of *n*-3 PUFA supplementation strategies for CVD prevention.

### 3.2. Incorporation of EPA and DHA into the Lipid Pool

EPA and DHA show differential metabolism-, tissue-, and compartment-specific accumulation following supplementation, which may affect their function. Supplementation with EPA and DHA leads to a dose-dependent increase in plasma, which reaches equilibrium approx. one month post-supplementation [78]. Studies suggest a very low conversion of EPA to DHA following supplementation. However, DHA supplementation increases plasma EPA concentrations, possibly due to retro-conversion or slow EPA turnover [78,79]. Following fish oil supplementation, EPA preferentially accumulates in cholesteryl esters within very low-density lipoproteins (VLDL), possibly due to increased selectivity of the enzyme lecithin-cholesterol acyltransferase to EPA [80,81,82]. On the other hand, DHA is a preferential substrate for diacylglycerol acyltransferase leading to increased incorporation into TG [81,82,83].

The source and formulation of *n*-3-PUFA supplements also affects their bioavailability and consequently their lipid incorporation. Studies comparing the incorporation of EPA and DHA into plasma lipids after short-term supplementation with nutritional or pharmacological doses (>3 g/day) of *n*-3-PUFAs, in the forms of free fatty acids, ethyl esters or re-esterified TG, have reported conflicting findings [84,85,86,87]. However, a clinical study investigating the effect of prolonged supplementation (6 months) of dyslipidemic patients on statins with moderate doses of *n*-3-PUFAs (1.01 g EPA and 0.67 g DHA), reported that the re-esterified triglyceride formulation showed higher incorporation into red blood cell membranes and a more effective reduction in fasting plasma TG, compared to the ethyl ester formulation [88,89]. The above study reiterates the importance of supplement formulation and length of treatment on its effectiveness.

### 3.3. Effects of EPA and DHA on Lipoprotein Metabolism

High levels of blood TG, transported by the TG-rich VLDLs and chylomicrons, increase the risk for atherosclerosis [90]. Dietary supplementation with fish oil or with pure EPA and DHA, attenuates plasma TG by inhibiting TG synthesis and VLDL production, and inducing apolipoprotein B degradation in hepatocytes [91,92,93,94]. Furthermore, *n*-3-PUFAs limit the supply of plasma non-esterified fatty acids for VLDL synthesis through inhibition of intracellular lipolysis in adipocytes [95,96]. Accelerated chylomicron clearance through induction of lipolysis has also been shown to be induced by both DHA and EPA [97]. A number of studies also demonstrate that *n*-3-PUFAs supplementation increases HDL levels; however, there are studies showing contradicting findings [65,98,99,100].

Studies using high doses of purified EPA and DHA, given separately or together, alone or in combination with statins, show a significant improvement of dyslipidemia [89,101,102]. Skulas-Ray et al., reported that supplementation with high doses (3.4 g/d) of EPA and DHA for eight weeks led to a significant reduction in plasma TG, whilst a lower dose (0.85 g/d) had no significant effect. Neither of the doses had any effect on endothelial function and inflammatory markers, possibly because the study subjects were healthy [102]. These findings suggest that high doses of purified *n*-3 PUFAs may be required to achieve optimum clinical efficacy.

However, a number of studies using EPA or DHA monotherapy report differential effects or efficacies of the two fatty acids on plasma lipids. The randomised cross-over study ComparED, compared the effect of DHA and EPA (2.7 g/day), formulated as re-esterified triacylglycerol, on blood lipids and inflammatory markers in subjects with abdominal obesity and subclinical inflammation. The study reported that DHA may be more effective in reducing TG, and increasing HDL- and LDL-cholesterol concentrations, compared to EPA. Apolipoprotein B levels were also reduced by DHA, but not by EPA [103]. A secondary analysis of the ComparED study results indicated that the increased efficacy of DHA in reducing TG does not depend on a greater effect magnitude but on the fact that a greater proportion of study subjects responded to DHA compared to EPA [104]. Previous studies also investigated the effect of supplementation with highly pure, free, or esterified, DHA or EPA (>3 g/day) for 4–7 weeks on the plasma lipid profile of healthy or hypertensive subjects. These studies report mixed findings, with some showing a superior effect of DHA compared to EPA supplementation and others no difference between the two *n*-3 PUFAs [97,105,106,107,108]. A meta-analysis of these studies concluded that overall DHA reduces TG and increases LDL and HDL cholesterol, to a greater extent than EPA [109]. High doses (3–4 g/day) of EPA or DHA were shown to be equally efficient in inducing liposome lipase activity, suggesting that the superior triglyceride-lowering effect of DHA is not due to a more efficient chylomicron/VLDL clearance [97,110].

Early studies have demonstrated that *n*-3 PUFAs reduce TG levels, at least partly, through inhibition of acyl CoA: diacylglycerol acyltransferase (DGAT), an enzyme catalysing the terminal step of TG synthesis [111]. *n*-3 PUFAs also act by inhibiting hepatic de novo synthesis of fatty acids and TG and inducing fatty acid oxidation and TG catabolism in adipose tissue and muscle [112]. These effects are mediated by modulating the activity of transcription factors, and more specifically the sterol regulatory element-binding protein (SREBP)-1 and the peroxisome proliferator-activated receptors (PPARs) [72]. SREBPs are cellular fatty acid sensors, which are activated by proteolytic release from the endoplasmic reticulum leading to their translocation to the nucleus, where they activate lipogenic gene transcription. SREB-1a and -1c isoforms are transcribed from the *SREBP-1* gene under the regulation of different promoters [113]. PUFAs, including *n*-3 PUFAs, inhibit SREBP-1 activation by preventing its proteolytic release from the ER, and reducing its gene expression by competing with its transcriptional activator heterodimer liver X receptor/retinoid X receptor (LXR/RXR) or by increasing its mRNA degradation [114,115,116]. RXR also form heterodimers with the nuclear receptors PPARs, which act as lipid sensors by binding to a number of different intracellular fatty acids species. PPARα, predominantly expressed in the liver, and PPARβ/δ that show higher expression in the skeletal and cardiac muscle, drive mitochondrial and peroxisomal fatty acid oxidation gene transcription. PPARγ is mainly found in adipose tissue where it regulates adipocyte differentiation and activity, including lipoprotein lipase activity and fatty acid oxidation. *n*-3 PUFAs act as ligands of RXR and all three PPAR isoforms, activating fatty acid oxidation and lipoprotein lipase gene expression [117,118,119].

Evidence from in vivo and in vitro studies suggests that EPA and DHA may differentially regulate these molecular transcriptional pathways. Studies in rat hepatocytes have shown that EPA is a more effective inhibitor of DGAT activity, compared to DHA. Furthermore, although EPA and DHA activate PPARα to a similar extent, EPA activates mitochondrial fatty acid oxidation, whilst DHA drives only peroxisomal fatty acid oxidation [83,120,121]. This is a result of differential substrate preference of each pathway, with EPA being oxidised both in microsomes and mitochondria, whereas DHA is only oxidised in microsomes [83] Based on these experimental findings, EPA may have a greater influence on TG levels, as mitochondrial fatty acid oxidation has a more significant influence on fatty acid availability for triglyceride synthesis, compared to microsomal oxidation [122]. Unfortunately, to the best of our knowledge, there are no published studies comparing the effects of EPA and DHA on fatty acid oxidation in humans.

Studies in rat models have shown that EPA and DHA inhibit lipogenesis both separately and in combination [123]. EPA has also been shown to inhibit LXR/RXR-SREBP-1c interaction more potently than DHA, in a human embryonic kidney cell line [114]. However, a randomised control trial, comparing the effects of DHA and EPA (3 g/day) on plasma triglyceride metabolism in young healthy subjects, reported that although both *n*-3-PUFAs inhibited liposome lipase activity, EPA increased the lipogenic index and failed to reduce plasma triglyceride levels. In contrast, DHA did not modulate the lipogenic index and significantly reduced triglyceride levels [110].

## 4. Specific PUFAs as Anti-Inflammatory and Anti-Oxidative Mediators

### 4.1. Effects of EPA and DHA on Inflammation

Another way through which *n*-3-PUFAs exert their protective effect against atherosclerosis, is by reducing vascular inflammation. Randomised control trials show that a mix of DHA and EPA in different proportions and formulations reduces plasma inflammatory markers, including C-reactive protein (CRP), IL-6 and TNFα, and increases the anti-inflammatory mediator adiponectin [124,125]. A recent meta-analysis of studies directly comparing the effects of DHA and EPA on plasma inflammation concluded that the two *n*-3-PUFAs do not have significantly differential effects on CRP, IL-6, TNFα and adiponectin [126].

*n*-3-PUFAs exert their anti-inflammatory effect by modulating the balance between pro- and anti-inflammatory lipid mediators. Following their release from phospholipids by phospholipases, PUFAs are metabolised by cyclooxygenases (COX), lipoxygenases (LOX) and cytochrome P450 (CYP450) into oxylipins that can act as endogenous mediators. *n*-6-PUFAs, such as arachidonic acid, act as pre-cursors for pro-inflammatory and pro-thrombotic eicosanoids, such as the 2-series prostaglandins and thromboxanes, and the 4-series leukotrienes. EPA gives rise to 3-series prostaglandins and thromboxanes and 5-series leukotrienes, which are less inflammatory. Furthermore, EPA and DHA promote inflammation resolution by being metabolised into anti-inflammatory mediators, such as resolvins derived from EPA and DHA, and protectins and maresins derived from DHA [127,128,129]. EPA and DHA promote an anti-inflammatory environment by competing with arachidonic acid for the metabolic enzyme sites, thus reducing the production of pro-inflammatory eicosanoids. Indeed, clinical studies have shown that moderate- or high-dose supplementation with EPA and DHA ethyl esters increases *n*-3-PUFA-derived anti-inflammatory oxylipins, whilst reducing *n*-6-derived eicosanoids in human plasma [130,131,132]. Differences in the metabolism of EPA and DHA can introduce variability in their clinical effects. CYP450 isoforms have differences with respect to their specificity for each *n*-3-PUFA, whilst the metabolism of EPA and DHA may also be affected by differences in CYP450 catalytic activity due to gene polymorphisms [133,134]. Therefore, tissue-dependent, and inter-individual differences in the expression and activities of CYP450 isoforms may lead to variation in the anti-inflammatory to EPA and DHA.

*n*-3-PUFAs also inhibit pro-inflammatory signaling pathways. Activation of PPARγ by DHA and EPA leads to inhibition of the pro-inflammatory transcription factor nuclear factor (NF)-κB and up-regulation of adiponectin production by adipocytes [135,136,137]. *n*-3-PUFAs also promote anti-inflammatory effects by binding to the G-protein coupled receptor Free fatty acid receptor 4 (FFAR4/GPR120), which is highly expressed in adipose tissue and macrophages. Upon binding to *n*-3-PUFAs, FFAR4 promotes calcium-dependent regulation of physiological processes, including glucose metabolism and adipogenesis, and recruits and activates the scaffold protein β-arrestin-2 that has anti-inflammatory effects [138]. Specifically, the FFAR4/β-arrestin-2 complex inhibits NF-κB activation and the formation of the NOD-like receptor protein 3 (NLRP3) inflammasome in macrophages. NLRP3 is a cytosolic protein complex that senses metabolic stress signals, such as cholesterol crystals, triggering the activation of the inflammatory cytokines IL-1β and IL-18 [139,140].

A number of in vitro studies in different cell types demonstrate that EPA and DHA differentially regulate inflammatory mechanisms. In the monocytic cell line THP-1, EPA and DHA were shown to inhibit NF-κB activity through two distinct signaling cascades [141]. Moreover, a study in the human colon carcinoma cell line CaCo-2 demonstrated that both EPA and DHA alone can trigger FFAR4-dependent inhibition of NF-κB with the same efficacy but with different kinetics, possibly due to different affinity to the receptor [142]. On the other hand, experiments in HT29, another colon carcinoma cell, line show that EPA activates both calcium and β-arrestin-2-mediated signaling, whilst DHA showed no calcium activation and weak β-arrestin-2 recruitment [143]. Human FFAR4 shows two alternative splice variants; the short form triggers both calcium- and arrestin-mediated cascades and the long form only drives arrestin signaling [144]. The relative responses of EPA and DHA on FFAR4 may therefore depend on the relative expression of each isoform, which can be cell line-, tissue- and/or disease-specific, and the relative affinity of each fatty acid for each isoform. In a rat model of myocardial infarction, EPA was shown to have a stronger anti-inflammatory effect compared to DHA, by maintaining a higher PPARγ activity through inhibition of its phosphorylation [145].

### 4.2. Effects of EPA and DHA on Lipid Oxidation

Dyslipidemia and oxidative stress promote oxidized (ox)LDL and cholesterol crystal domain formation in endothelial cell membranes, which promote endothelial dysfunction and vascular inflammation. OxLDL triggers endothelial activation and the recruitment of macrophages leading to the formation of foam cells, which promote inflammation and atherosclerotic plague destabilisation [146]. Cholesterol crystal domains promote inflammation by activating the NLRP3 inflammasome, a protein complex that triggers caspase-induced activation of the cytokines IL-1β and IL-18 [147]. Both EPA and DHA, and their oxidation products, protect against LDL and cell membrane oxidation by directly scavenging reactive oxygen species, and by increasing endogenous antioxidant gene expression through activation of the cytoprotective transcription factor nuclear factor erythroid 2–related factor 2 (Nrf2) [148,149,150,151]. EPA was shown to have a stronger and more prolonged protective effect against LDL oxidation and cholesterol crystal domain formation, compared to DHA, in in vitro studies. This may be due to the chemical structure of EPA, and specifically the combination of its hydrocarbon length and location of double bonds, which allows more efficient electron stabilization [152,153].

### 4.3. Effects of EPA and DHA on Cell Death

Evidence supports the protective effects of *n*-3 PUFAs against apoptosis and cell death in atherosclerosis. EPA/DHA-rich oxLDL obtained from healthy subjects following fish oil supplementation, was shown to trigger less apoptosis in a monocytic cell line, compared to control oxLDL [154]. Furthermore, DHA supplementation (400–1600 mg/day) of healthy subjects for two weeks was shown to dose-dependently reduce the susceptibility of peripheral blood monocytes to oxLDL-dependent apoptosis [155]. In line with these clinical observations, in vitro studies report that oxidised EPA and DHA promote monocyte-derived macrophage apoptosis, but to a lesser extent than oxidised arachidonic acid, whilst DHA inhibits saturated fatty acid-induced endothelial cell apoptosis [156,157]. Maresin-1, a DHA metabolite, also inhibits endothelial cell inflammatory responses and apoptosis by activating PPARα-mediated signaling [158].

A study in an obese mouse model showed that an imbalance in saturated fatty acid/*n*-3 PUFA levels in peritoneal macrophage membranes is associated with impaired removal of apoptotic cells by efferocytosis, due to altered phosphatidylinositol 3-kinase activity. Intriguingly, the same study showed that fish oil supplementation normalised efferocytosis, indicating a role of *n*-3 PUFAs in effective clearance of apoptotic cells [159]. Indeed, DHA was shown to enhance the efferocytosis activity of macrophages by driving their differentiation to a more anti-inflammatory and pro-resolving phenotype via a PPARγ-dependent mechanism [160]. The DHA metabolite Resolvin D1 also promotes the efferocytosis of necroptotic cells by promoting cytoskeletal changes, and mitochondrial respiration and ATP production [161,162].

The above studies show the complexity of the molecular mechanisms underlying the effects of EPA and DHA on lipoprotein metabolism and oxidation, and inflammation. This is further compounded by the heterogeneity of the in vitro and in vivo models used, due to differences in experimental conditions, and tissue- and species-specific differences in signaling pathways. Furthermore, these models do not accurately represent the in vivo situation, where there is interaction between multiple cells, such as between immune cells with endothelial cells and adipocytes. Development of more reproducible and accurate models would enable a better understanding of mechanisms of action of EPA and DHA in order to determine their relative efficacies and optimise supplementation composition.

## 5. Epigenetic Determinants of *n*-3-PUFA Effects

An interesting observation from the ComparED study is that only 26% of subjects responded to either DHA or EPA. As the compliance of the study subjects was approximately 95%, the lack of response was probably not due to non-compliance but due to other factors [104]. This suggests that the variability observed in clinical studies could be a result of inter-individual differences in the response to *n*-3 PUFAs. These differences may stem from genomic differences, as a number of single nucleotide polymorphisms (SNPs) in genes involved in lipid metabolism and triglyceride synthesis have been associated with the effect of *n*-3 PUFAs on plasma TG [163,164,165,166]. Epigenetic mechanisms may also contribute to the variability in the response to *n*-3 PUFAs.

Availability of *n*-3 PUFAs, particularly DHA, is required for one-carbon metabolism that leads to the production of S-adenosyl methionine, the methyl donor of DNA methylation [167]. Indeed, a number of clinical studies in the last 10 years have demonstrated an effect of *n*-3 PUFAs on DNA methylation. DNA methylation analysis on blood from Yupik natives identified 27 differentially-methylated CpG sites that correlated with the levels of *n*-3 PUFA intake, including the fatty acid synthase gene, and the aryl-hydrocarbon receptor repressor gene involved in antioxidant protection [168]. Furthermore, a study in lactating infants and adult men reported a strong association between EPA and arachidonic acid levels with global DNA methylation, with EPA having a stronger association than arachidonic acid [169]. A randomised control study in healthy or chronic kidney disease patients showed that 4 g/day *n*-3 PUFAs (1.8 g EPA, 0.2 g DPA, 1.5 g DHA ethyl esters) for 8 weeks, led to altered CpG methylation in the 5′-regulatory region of fatty acid desaturase *(FADS) 2* gene, encoding Δ6 desaturase, and elongase *(ELOVL)-5* that encodes elongase 5, in peripheral blood mononuclear cells (PBMCs) [170]. A study conducted in PBMCs from obese subjects on a low-calorie diet found that supplementation with *n*-3 PUFA-rich fish oil for 8 weeks altered CpG methylation at a specific site in the *CD36* gene, a membrane glycoprotein that is involved in lipid uptake, including oxLDL internalisation, in macrophages. Nonetheless, the changes in methylation reported were very small suggesting that they may not affect gene expression [171]. Studies have also investigated the effects of prenatal *n*-3 PUFA supplementation on the infant epigenome.

Supplementation of pregnant women with DHA (400 mg/day) from gestation week 18–22 until birth was associated with increased methylation of the imprinted genes insulin-like growth factor 2 (*IGF2*) and *H19* in umbilical cord mononuclear cells, compared to the control group [172]. *IGF2*/*H19* differentially methylated regions have been associated with the risk of higher birth and early life weight [173,174]. A subsequent randomised control study reported that supplementation of pregnant women with DHA-rich fish oil (800 mg/day) from gestational week 20 until delivery did not significantly alter global DNA methylation in blood leukocytes, but induced small changes in the methylation of a subset of genes involved in lipid metabolism, appetite regulation and immune function [175]. Similarly, a study on 9-month-old infants supplemented with EPA/DHA-rich fish oil (1.6 g/day) for 9 months, showed no statistically-significant difference in global DNA methylation [176].

The above studies show that *n*-3 PUFAs may promote their clinical effects through epigenomic re-programming. However, the findings show small changes in DNA methylation, which may not have important functional consequences. This may be due to the small sample size of the studies or to insufficient *n*-3 PUFAs dose. Furthermore, as DNA methylation patterns are tissue-specific, epigenetic analysis of other tissues may lead to a more complete picture of the epigenetic effects of *n*-3 PUFAs. Importantly, functional studies are required in order to understand the clinical implications of these changes.

## 6. Conclusions and Outlook

CVD is the leading cause of mortality worldwide [68]. It is more than clear nowadays that *n*-3 PUFAs do not only serve as an inert form of energy storage [177]. Epidemiological studies and randomized control trials have reported that *n*-3 PUFAs might reduce cardiovascular events [54]. The evidence of the benefits of *n*-3 PUFAs is stronger in secondary than in primary prevention settings [34]. Several potential mechanisms for the cardioprotective effect of *n*-3 PUFAs have been proposed, with the effect on blood lipids being the most scientifically proven [44,45]. Particularly, their most consistent effect is the reduction in serum TG [54]. Nonetheless, there are conflicting findings regarding their clinical benefits, leading to uncertainty regarding their use for preventing atherosclerosis [26]. Several factors need to be considered when designing clinical studies, especially when natural ingredients and natural extracts are tested for efficacy. Firstly, as the TG-lowering effects of *n*-3 PUFA are dose-dependent, the use of consistent, and preferably high doses, across studies may lead to more meaningful results [102]. Secondly, the selection of suitable study subjects is crucial as *n*-3 PUFAs were shown to have a more pronounced effect in patients with hypertriglyceridaemia [61]. Thirdly, the selection of the chemical form, composition, and EPA/DHA ratio of *n*-3 PUFA is an important factor, as it affects their overall molecular effects. Another important factor in accurately collecting data is the consideration of the run-in period that is about 3 to 6 months from the time of the supplement first consumed. A period that is known for some natural products as required to indicate activity.

Optimising the EPA and DHA content of *n*-3 PUFA supplements requires a clear understanding of the molecular mechanisms driving their effects. Currently most of the available mechanistic data have been obtained from in vitro and animal models, whilst very limited data from humans are available [178]. Moving towards 3-dimentional organoid cultures of patient-derived cells would permit studying the effects of *n*-3 PUFAs in an environment that more closely resembles the human in vivo situation. This approach will generate data that can be more easily translated into clinical studies.

Another important issue is identifying or predicting which patients respond to *n*-3 PUFA supplementation in order to provide more personalised management. More extensive and better-powered epigenomic studies, accompanied by functional studies, would elucidate the role of epigenetic mechanisms in the cellular effects of *n*-3 PUFAs, as well as epigenetic biomarkers for predicting responders or non-responders. In line with this notion, transcriptomics analysis of PBMCs of responders or non-responders to the TG-lowering effect of EPA/DHA-rich fish oil (1.6 g/day), identified differentially regulated genes between the two groups [179]. Future studies could use transcriptomics and metabolomics analysis to identify gene and metabolite signatures, which could be used as biomarkers for predicting patients that would benefit from the use of *n*-3 PUFAs. This could involve performing DNA methylation, transcriptomics and/or metabolomics analysis of blood plasma samples before and after treatment with *n*-3 PUFAs supplements. The potential biomarkers could then be correlated with blood lipids, and inflammatory and thrombotic markers, and their predictive accuracy determined. Furthermore, integration of transcriptomics and metabolomics data can be used in order to identify new *n*-3-PUFA-regulated pathways, which could lead to a better understanding of the molecular mechanisms underlying their effects.

Concluding, the existing clinical trials are not appropriately designed considering all important parameters to correctly evaluate their efficacy in the treatment of different CVDs. High doses and ratio with DHA in excess might be the answer to the mystery. All the rational and biochemical networks that can be affected by the use of these natural molecules as supplements suggest the need for carefully designed basic experimental studies and clinical trials in order to reach accurate conclusions on their efficacy in treating different CVDs.

## References

[1] Frostegard J. (2013). Immunity, atherosclerosis and cardiovascular disease. BMC Med..

[2] Libby P. (2002). Inflammation in atherosclerosis. Nature.

[3] Thom T., Haase N., Rosamond W., Howard V.J., Rumsfeld J., Manolio T., Zheng Z.J., Flegal K., O’Donnell C., Kittner S. (2006). Heart disease and stroke statistics—2006 update: A report from the American Heart Association Statistics Committee and Stroke Statistics Subcommittee. Circulation.

[4] Reimer K.A., Jennings R.B., Tatum A.H. (1983). Pathobiology of acute myocardial ischemia: Metabolic, functional and ultrastructural studies. Am. J. Cardiol..

[5] Hajouli S., Ludhwani D. (2023). Heart Failure and Ejection Fraction. StatPearls.

[6] Gasbarrino K., Di Iorio D., Daskalopoulou S.S. (2022). Importance of sex and gender in ischaemic stroke and carotid atherosclerotic disease. Eur. Heart J..

[7] Mohd Nor N.S., Al-Khateeb A.M., Chua Y.A., Mohd Kasim N.A., Mohd Nawawi H. (2019). Heterozygous familial hypercholesterolaemia in a pair of identical twins: A case report and updated review. BMC Pediatr..

[8] Pahwa R., Jialal I. (2023). Atherosclerosis. StatPearls.

[9] Dichgans M., Pulit S.L., Rosand J. (2019). Stroke genetics: Discovery, biology, and clinical applications. Lancet Neurol..

[10] Sakakura K., Nakano M., Otsuka F., Ladich E., Kolodgie F.D., Virmani R. (2013). Pathophysiology of atherosclerosis plaque progression. Heart Lung Circ..

[11] Kowara M., Cudnoch-Jedrzejewska A. (2021). Pathophysiology of Atherosclerotic Plaque Development-Contemporary Experience and New Directions in Research. Int. J. Mol. Sci..

[12] Libby P., Buring J.E., Badimon L., Hansson G.K., Deanfield J., Bittencourt M.S., Tokgozoglu L., Lewis E.F. (2019). Atherosclerosis. Nat. Rev. Dis. Primers.

[13] Galis Z.S., Sukhova G.K., Lark M.W., Libby P. (1994). Increased expression of matrix metalloproteinases and matrix degrading activity in vulnerable regions of human atherosclerotic plaques. J. Clin. Investig..

[14] Amento E.P., Ehsani N., Palmer H., Libby P. (1991). Cytokines and growth factors positively and negatively regulate interstitial collagen gene expression in human vascular smooth muscle cells. Arterioscler. Thromb..

[15] Galis Z.S., Sukhova G.K., Kranzhofer R., Clark S., Libby P. (1995). Macrophage foam cells from experimental atheroma constitutively produce matrix-degrading proteinases. Proc. Natl. Acad. Sci. USA.

[16] Lin J., Li H., Yang M., Ren J., Huang Z., Han F., Huang J., Ma J., Zhang D., Zhang Z. (2013). A role of RIP3-mediated macrophage necrosis in atherosclerosis development. Cell Rep..

[17] Geng Y.J., Libby P. (1995). Evidence for apoptosis in advanced human atheroma. Colocalization with interleukin-1 beta-converting enzyme. Am. J. Pathol..

[18] Schrijvers D.M., De Meyer G.R., Kockx M.M., Herman A.G., Martinet W. (2005). Phagocytosis of apoptotic cells by macrophages is impaired in atherosclerosis. Arterioscler. Thromb. Vasc. Biol..

[19] Spannella F., Giulietti F., Di Pentima C., Sarzani R. (2019). Prevalence and Control of Dyslipidemia in Patients Referred for High Blood Pressure: The Disregarded “Double-Trouble” Lipid Profile in Overweight/Obese. Adv. Ther..

[20] Esper R.J., Nordaby R.A. (2019). Cardiovascular events, diabetes and guidelines: The virtue of simplicity. Cardiovasc. Diabetol..

[21] Arnett D.K., Blumenthal R.S., Albert M.A., Buroker A.B., Goldberger Z.D., Hahn E.J., Himmelfarb C.D., Khera A., Lloyd-Jones D., McEvoy J.W. (2019). 2019 ACC/AHA Guideline on the Primary Prevention of Cardiovascular Disease: A Report of the American College of Cardiology/American Heart Association Task Force on Clinical Practice Guidelines. Circulation.

[22] Puttananjaiah M.K., Dhale M.A., Gaonkar V., Keni S. (2011). Statins: 3-hydroxy-3-methylglutaryl-CoA (HMG-CoA) reductase inhibitors demonstrate anti-atherosclerotic character due to their antioxidant capacity. Appl. Biochem. Biotechnol..

[23] Li E.C., Heran B.S., Wright J.M. (2014). Angiotensin converting enzyme (ACE) inhibitors versus angiotensin receptor blockers for primary hypertension. Cochrane Database Syst. Rev..

[24] Misra S., Stevermer J.J. (2009). ACE inhibitors and ARBs: One or the other--not both--for high-risk patients. J. Fam. Pract..

[25] Sikand G., Severson T. (2020). Top 10 dietary strategies for atherosclerotic cardiovascular risk reduction. Am. J. Prev. Cardiol..

[26] Yagi S., Fukuda D., Aihara K.I., Akaike M., Shimabukuro M., Sata M. (2017). *n*-3 Polyunsaturated Fatty Acids: Promising Nutrients for Preventing Cardiovascular Disease. J. Atheroscler. Thromb..

[27] Deckelbaum R.J., Worgall T.S., Seo T. (2006). *n*-3 fatty acids and gene expression. Am. J. Clin. Nutr..

[28] Deckelbaum R.J., Akabas S.R. (2006). *n*-3 Fatty acids and cardiovascular disease: Navigating toward recommendations. Am. J. Clin. Nutr..

[29] Sullivan E.J. (1996). Education. Is tenure still viable today?. J. Prof. Nurs..

[30] Sokola-Wysoczanska E., Wysoczanski T., Wagner J., Czyz K., Bodkowski R., Lochynski S., Patkowska-Sokola B. (2018). Polyunsaturated Fatty Acids and Their Potential Therapeutic Role in Cardiovascular System Disorders—A Review. Nutrients.

[31] Simopoulos A.P. (2008). The omega-6/omega-3 fatty acid ratio, genetic variation, and cardiovascular disease. Asia Pac. J. Clin. Nutr..

[32] Zhao G., Etherton T.D., Martin K.R., Gillies P.J., West S.G., Kris-Etherton P.M. (2007). Dietary alpha-linolenic acid inhibits proinflammatory cytokine production by peripheral blood mononuclear cells in hypercholesterolemic subjects. Am. J. Clin. Nutr..

[33] Eslick G.D., Howe P.R., Smith C., Priest R., Bensoussan A. (2009). Benefits of fish oil supplementation in hyperlipidemia: A systematic review and meta-analysis. Int. J. Cardiol..

[34] Marik P.E., Varon J. (2009). Omega-3 dietary supplements and the risk of cardiovascular events: A systematic review. Clin. Cardiol..

[35] Mohan D., Mente A., Dehghan M., Rangarajan S., O’Donnell M., Hu W., Dagenais G., Wielgosz A., Lear S., Wei L. (2021). Associations of Fish Consumption With Risk of Cardiovascular Disease and Mortality Among Individuals With or Without Vascular Disease From 58 Countries. JAMA Intern. Med..

[36] Shahidi F., Ambigaipalan P. (2018). Omega-3 Polyunsaturated Fatty Acids and Their Health Benefits. Annu. Rev. Food Sci. Technol..

[37] Mariamenatu A.H., Abdu E.M. (2021). Overconsumption of Omega-6 Polyunsaturated Fatty Acids (PUFAs) versus Deficiency of Omega-3 PUFAs in Modern-Day Diets: The Disturbing Factor for Their “Balanced Antagonistic Metabolic Functions” in the Human Body. J. Lipids.

[38] Barceló-Coblijn G., Murphy E.J. (2009). Alpha-linolenic acid and its conversion to longer chain *n*-3 fatty acids: Benefits for human health and a role in maintaining tissue *n*-3 fatty acid levels. Prog. Lipid Res..

[39] Bird J.K., Calder P.C., Eggersdorfer M. (2018). The Role of *n*-3 Long Chain Polyunsaturated Fatty Acids in Cardiovascular Disease Prevention, and Interactions with Statins. Nutrients.

[40] Metcalf R.G., James M.J., Gibson R.A., Edwards J.R., Stubberfield J., Stuklis R., Roberts-Thomson K., Young G.D., Cleland L.G. (2007). Effects of fish-oil supplementation on myocardial fatty acids in humans. Am. J. Clin. Nutr..

[41] Watanabe Y., Tatsuno I. (2020). Prevention of Cardiovascular Events with Omega-3 Polyunsaturated Fatty Acids and the Mechanism Involved. J. Atheroscler. Thromb..

[42] Russo G.L. (2009). Dietary *n*-6 and *n*-3 polyunsaturated fatty acids: From biochemistry to clinical implications in cardiovascular prevention. Biochem. Pharmacol..

[43] Massaro M., Scoditti E., Carluccio M.A., De Caterina R. (2008). Basic mechanisms behind the effects of *n*-3 fatty acids on cardiovascular disease. Prostaglandins Leukot. Essent. Fat. Acids.

[44] Lavie C.J., Milani R.V., Mehra M.R., Ventura H.O. (2009). Omega-3 polyunsaturated fatty acids and cardiovascular diseases. J. Am. Coll. Cardiol..

[45] Balk E.M., Lichtenstein A.H., Chung M., Kupelnick B., Chew P., Lau J. (2006). Effects of omega-3 fatty acids on serum markers of cardiovascular disease risk: A systematic review. Atherosclerosis.

[46] Liu Y.X., Yu J.H., Sun J.H., Ma W.Q., Wang J.J., Sun G.J. (2023). Effects of Omega-3 Fatty Acids Supplementation on Serum Lipid Profile and Blood Pressure in Patients with Metabolic Syndrome: A Systematic Review and Meta-Analysis of Randomized Controlled Trials. Foods.

[47] Kones R., Howell S., Rumana U. (2017). *n*-3 Polyunsaturated Fatty Acids and Cardiovascular Disease: Principles, Practices, Pitfalls, and Promises—A Contemporary Review. Med. Princ. Pract. Int. J. Kuwait Univ. Health Sci. Cent..

[48] Farnier M., Zeller M., Masson D., Cottin Y. (2021). Triglycerides and risk of atherosclerotic cardiovascular disease: An update. Arch. Cardiovasc. Dis..

[49] Kelley D.S., Siegel D., Vemuri M., Mackey B.E. (2007). Docosahexaenoic acid supplementation improves fasting and postprandial lipid profiles in hypertriglyceridemic men. Am. J. Clin. Nutr..

[50] Harper C.R., Edwards M.C., Jacobson T.A. (2006). Flaxseed oil supplementation does not affect plasma lipoprotein concentration or particle size in human subjects. J. Nutr..

[51] Harris W.S., Von Schacky C. (2004). The Omega-3 Index: A new risk factor for death from coronary heart disease?. Prev. Med..

[52] Harris W.S., Zotor F.B. (2019). *n*-3 Fatty acids and risk for fatal coronary disease. Proc. Nutr. Soc..

[53] Milte C.M., Coates A.M., Buckley J.D., Hill A.M., Howe P.R. (2008). Dose-dependent effects of docosahexaenoic acid-rich fish oil on erythrocyte docosahexaenoic acid and blood lipid levels. Br. J. Nutr..

[54] Torrejon C., Jung U.J., Deckelbaum R.J. (2007). *n*-3 Fatty acids and cardiovascular disease: Actions and molecular mechanisms. Prostaglandins Leukot. Essent. Fat. Acids.

[55] BNF (2005). Cardiovascular Disease: Diet, Nutrition and Emerging Risk Factors.

[56] Breslow J.L. (2006). *n*-3 fatty acids and cardiovascular disease. Am. J. Clin. Nutr..

[57] Yusof H.M., Miles E.A., Calder P. (2008). Influence of very long-chain *n*-3 fatty acids on plasma markers of inflammation in middle-aged men. Prostaglandins Leukot. Essent. Fat. Acids.

[58] Sherratt S.C.R., Libby P., Budoff M.J., Bhatt D.L., Mason R.P. (2023). Role of Omega-3 Fatty Acids in Cardiovascular Disease: The Debate Continues. Curr. Atheroscler. Rep..

[59] SACN (2004). Advice on Fish Consumption: Benefits and Risks.

[60] Toth P.P., Chapman M.J., Parhofer K.G., Nelson J.R. (2022). Differentiating EPA from EPA/DHA in cardiovascular risk reduction. Am. Heart J. Plus Cardiol. Res. Pract..

[61] Geppert J., Kraft V., Demmelmair H., Koletzko B. (2006). Microalgal docosahexaenoic acid decreases plasma triacylglycerol in normolipidaemic vegetarians: A randomised trial. Br. J. Nutr..

[62] Skulas-Ray A.C., Wilson P.W.F., Harris W.S., Brinton E.A., Kris-Etherton P.M., Richter C.K., Jacobson T.A., Engler M.B., Miller M., Robinson J.G. (2019). Omega-3 Fatty Acids for the Management of Hypertriglyceridemia: A Science Advisory From the American Heart Association. Circulation.

[63] FDA (2004). Letter Responding to Health Claim Petition dated November 3, 2003 (MartekPetition): Omega-3 Fatty Acids and Reduced Risk of Coronary Heart Disease. http://www.cfsan.fda.gov/~dms/ds-ltr37.html.

[64] Chary A., Tohidi M., Hedayati M. (2023). Association of LDL-cholesterol subfractions with cardiovascular disorders: A systematic review. BMC Cardiovasc. Disord..

[65] Buckley R., Shewring B., Turner R., Yaqoob P., Minihane A.M. (2004). Circulating triacylglycerol and apoE levels in response to EPA and docosahexaenoic acid supplementation in adult human subjects. Br. J. Nutr..

[66] Wang H.H., Garruti G., Liu M., Portincasa P., Wang D.Q. (2017). Cholesterol and Lipoprotein Metabolism and Atherosclerosis: Recent Advances In reverse Cholesterol Transport. Ann. Hepatol..

[67] Innes J.K., Calder P.C. (2018). The Differential Effects of Eicosapentaenoic Acid and Docosahexaenoic Acid on Cardiometabolic Risk Factors: A Systematic Review. Int. J. Mol. Sci..

[68] Kelley D.S., Siegel D., Vemuri M., Chung G.H., Mackey B.E. (2008). Docosahexaenoic acid supplementation decreases remnant-like particle-cholesterol and increases the (*n*-3) index in hypertriglyceridemic men. J. Nutr..

[69] Harris W.S. (2007). Omega-3 fatty acids and cardiovascular disease: A case for omega-3 index as a new risk factor. Pharmacol. Res..

[70] Schuchardt J.P., Ostermann A.I., Stork L., Kutzner L., Kohrs H., Greupner T., Hahn A., Schebb N.H. (2016). Effects of docosahexaenoic acid supplementation on PUFA levels in red blood cells and plasma. Prostaglandins Leukot. Essent. Fat. Acids.

[71] Walker C.G., West A.L., Browning L.M., Madden J., Gambell J.M., Jebb S.A., Calder P.C. (2015). The Pattern of Fatty Acids Displaced by EPA and DHA Following 12 Months Supplementation Varies between Blood Cell and Plasma Fractions. Nutrients.

[72] Jump D.B. (2002). The biochemistry of *n*-3 polyunsaturated fatty acids. J. Biol. Chem..

[73] Yokoyama M., Origasa H., Matsuzaki M., Matsuzawa Y., Saito Y., Ishikawa Y., Oikawa S., Sasaki J., Hishida H., Itakura H. (2007). Effects of eicosapentaenoic acid on major coronary events in hypercholesterolaemic patients (JELIS): A randomised open-label, blinded endpoint analysis. Lancet.

[74] Bhatt D.L., Steg P.G., Miller M., Brinton E.A., Jacobson T.A., Ketchum S.B., Doyle R.T., Juliano R.A., Jiao L., Granowitz C. (2019). Cardiovascular Risk Reduction with Icosapent Ethyl for Hypertriglyceridemia. N. Engl. J. Med..

[75] Group A.S.C., Bowman L., Mafham M., Wallendszus K., Stevens W., Buck G., Barton J., Murphy K., Aung T., Haynes R. (2018). Effects of *n*-3 Fatty Acid Supplements in Diabetes Mellitus. N. Engl. J. Med..

[76] Manson J.E., Cook N.R., Lee I.M., Christen W., Bassuk S.S., Mora S., Gibson H., Albert C.M., Gordon D., Copeland T. (2019). Marine *n*-3 Fatty Acids and Prevention of Cardiovascular Disease and Cancer. N. Engl. J. Med..

[77] Sweeney T.E., Gaine S.P., Michos E.D. (2023). Eicosapentaenoic acid vs. docosahexaenoic acid for the prevention of cardiovascular disease. Curr. Opin. Endocrinol. Diabetes Obes..

[78] Arterburn L.M., Hall E.B., Oken H. (2006). Distribution, interconversion, and dose response of *n*-3 fatty acids in humans. Am. J. Clin. Nutr..

[79] Metherel A.H., Irfan M., Klingel S.L., Mutch D.M., Bazinet R.P. (2019). Compound-specific isotope analysis reveals no retroconversion of DHA to EPA but substantial conversion of EPA to DHA following supplementation: A randomized control trial. Am. J. Clin. Nutr..

[80] Singer P., Berger I., Wirth M., Godicke W., Jaeger W., Voigt S. (1986). Slow desaturation and elongation of linoleic and alpha-linolenic acids as a rationale of eicosapentaenoic acid-rich diet to lower blood pressure and serum lipids in normal, hypertensive and hyperlipemic subjects. Prostaglandins Leukot. Med..

[81] Subbaiah P.V., Kaufman D., Bagdade J.D. (1993). Incorporation of dietary *n*-3 fatty acids into molecular species of phosphatidyl choline and cholesteryl ester in normal human plasma. Am. J. Clin. Nutr..

[82] Sanders T.A., Sullivan D.R., Reeve J., Thompson G.R. (1985). Triglyceride-lowering effect of marine polyunsaturates in patients with hypertriglyceridemia. Arteriosclerosis.

[83] Madsen L., Rustan A.C., Vaagenes H., Berge K., Dyroy E., Berge R.K. (1999). Eicosapentaenoic and docosahexaenoic acid affect mitochondrial and peroxisomal fatty acid oxidation in relation to substrate preference. Lipids.

[84] Nordoy A., Barstad L., Connor W.E., Hatcher L. (1991). Absorption of the *n*-3 eicosapentaenoic and docosahexaenoic acids as ethyl esters and triglycerides by humans. Am. J. Clin. Nutr..

[85] Krokan H.E., Bjerve K.S., Mork E. (1993). The enteral bioavailability of eicosapentaenoic acid and docosahexaenoic acid is as good from ethyl esters as from glyceryl esters in spite of lower hydrolytic rates by pancreatic lipase in vitro. Biochim. Biophys. Acta.

[86] Dyerberg J., Madsen P., Moller J.M., Aardestrup I., Schmidt E.B. (2010). Bioavailability of marine *n*-3 fatty acid formulations. Prostaglandins Leukot. Essent. Fat. Acids.

[87] Hedengran A., Szecsi P.B., Dyerberg J., Harris W.S., Stender S. (2015). *n*-3 PUFA esterified to glycerol or as ethyl esters reduce non-fasting plasma triacylglycerol in subjects with hypertriglyceridemia: A randomized trial. Lipids.

[88] Neubronner J., Schuchardt J.P., Kressel G., Merkel M., von Schacky C., Hahn A. (2011). Enhanced increase of omega-3 index in response to long-term *n*-3 fatty acid supplementation from triacylglycerides versus ethyl esters. Eur. J. Clin. Nutr..

[89] Schuchardt J.P., Neubronner J., Kressel G., Merkel M., von Schacky C., Hahn A. (2011). Moderate doses of EPA and DHA from re-esterified triacylglycerols but not from ethyl-esters lower fasting serum triacylglycerols in statin-treated dyslipidemic subjects: Results from a six month randomized controlled trial. Prostaglandins Leukot. Essent. Fat. Acids.

[90] Gabani M., Shapiro M.D., Toth P.P. (2023). The Role of Triglyceride-rich Lipoproteins and Their Remnants in Atherosclerotic Cardiovascular Disease. Eur. Cardiol..

[91] Rustan A.C., Nossen J.O., Christiansen E.N., Drevon C.A. (1988). Eicosapentaenoic acid reduces hepatic synthesis and secretion of triacylglycerol by decreasing the activity of acyl-coenzyme A:1,2-diacylglycerol acyltransferase. J. Lipid Res..

[92] Wang H., Chen X., Fisher E.A. (1993). *n*-3 fatty acids stimulate intracellular degradation of apoprotein B in rat hepatocytes. J. Clin. Investig..

[93] Bordin P., Bodamer O.A., Venkatesan S., Gray R.M., Bannister P.A., Halliday D. (1998). Effects of fish oil supplementation on apolipoprotein B100 production and lipoprotein metabolism in normolipidaemic males. Eur. J. Clin. Nutr..

[94] Yuan M., Zhang Y., Hua T., Liu X.L., Liu T., Yuan R.Y., Li G.P., Zhu Y., Zhang X. (2021). Omega-3 polyunsaturated fatty acid supplementation improves lipid metabolism and endothelial function by providing a beneficial eicosanoid-pattern in patients with acute myocardial infarction: A randomized, controlled trial. Clin. Nutr..

[95] Rustan A.C., Hustvedt B.E., Drevon C.A. (1993). Dietary supplementation of very long-chain *n*-3 fatty acids decreases whole body lipid utilization in the rat. J. Lipid Res..

[96] Lorente-Cebrian S., Bustos M., Marti A., Fernandez-Galilea M., Martinez J.A., Moreno-Aliaga M.J. (2012). Eicosapentaenoic acid inhibits tumour necrosis factor-alpha-induced lipolysis in murine cultured adipocytes. J. Nutr. Biochem..

[97] Park Y., Harris W.S. (2003). Omega-3 fatty acid supplementation accelerates chylomicron triglyceride clearance. J. Lipid Res..

[98] Chen H., Deng G., Zhou Q., Chu X., Su M., Wei Y., Li L., Zhang Z. (2020). Effects of eicosapentaenoic acid and docosahexaenoic acid versus alpha-linolenic acid supplementation on cardiometabolic risk factors: A meta-analysis of randomized controlled trials. Food Funct..

[99] Borja-Magno A., Guevara-Cruz M., Flores-Lopez A., Carrillo-Dominguez S., Granados J., Arias C., Perry M., Sears B., Bourges H., Gomez F.E. (2023). Differential effects of high dose omega-3 fatty acids on metabolism and inflammation in patients with obesity: Eicosapentaenoic and docosahexaenoic acid supplementation. Front. Nutr..

[100] Hande L.N., Kjellmo C., Pettersen K., Ljunggren S., Karlsson H., Cederbrant K., Marcusson-Stahl M., Hovland A., Lappegard K.T. (2022). Effect of *n*-3 Polyunsaturated Fatty Acids on Lipid Composition in Familial Hypercholesterolemia: A Randomized Crossover Trial. Biomedicines.

[101] Pena-de-la-Sancha P., Munoz-Garcia A., Espinola-Zavaleta N., Bautista-Perez R., Mejia A.M., Luna-Luna M., Lopez-Olmos V., Rodriguez-Perez J.M., Fragoso J.M., Carreon-Torres E. (2023). Eicosapentaenoic and Docosahexaenoic Acid Supplementation Increases HDL Content in *n*-3 Fatty Acids and Improves Endothelial Function in Hypertriglyceridemic Patients. Int. J. Mol. Sci..

[102] Skulas-Ray A.C., Kris-Etherton P.M., Harris W.S., Vanden Heuvel J.P., Wagner P.R., West S.G. (2011). Dose-response effects of omega-3 fatty acids on triglycerides, inflammation, and endothelial function in healthy persons with moderate hypertriglyceridemia. Am. J. Clin. Nutr..

[103] Allaire J., Couture P., Leclerc M., Charest A., Marin J., Lepine M.C., Talbot D., Tchernof A., Lamarche B. (2016). A randomized, crossover, head-to-head comparison of eicosapentaenoic acid and docosahexaenoic acid supplementation to reduce inflammation markers in men and women: The Comparing EPA to DHA (ComparED) Study. Am. J. Clin. Nutr..

[104] Allaire J., Vors C., Harris W.S., Jackson K.H., Tchernof A., Couture P., Lamarche B. (2019). Comparing the serum TAG response to high-dose supplementation of either DHA or EPA among individuals with increased cardiovascular risk: The ComparED study. Br. J. Nutr..

[105] Egert S., Kannenberg F., Somoza V., Erbersdobler H.F., Wahrburg U. (2009). Dietary alpha-linolenic acid, EPA, and DHA have differential effects on LDL fatty acid composition but similar effects on serum lipid profiles in normolipidemic humans. J. Nutr..

[106] Grimsgaard S., Bonaa K.H., Hansen J.B., Nordoy A. (1997). Highly purified eicosapentaenoic acid and docosahexaenoic acid in humans have similar triacylglycerol-lowering effects but divergent effects on serum fatty acids. Am. J. Clin. Nutr..

[107] Nestel P., Shige H., Pomeroy S., Cehun M., Abbey M., Raederstorff D. (2002). The *n*-3 fatty acids eicosapentaenoic acid and docosahexaenoic acid increase systemic arterial compliance in humans. Am. J. Clin. Nutr..

[108] Woodman R.J., Mori T.A., Burke V., Puddey I.B., Watts G.F., Beilin L.J. (2002). Effects of purified eicosapentaenoic and docosahexaenoic acids on glycemic control, blood pressure, and serum lipids in type 2 diabetic patients with treated hypertension. Am. J. Clin. Nutr..

[109] Wei M.Y., Jacobson T.A. (2011). Effects of eicosapentaenoic acid versus docosahexaenoic acid on serum lipids: A systematic review and meta-analysis. Curr. Atheroscler. Rep..

[110] Klingel S.L., Metherel A.H., Irfan M., Rajna A., Chabowski A., Bazinet R.P., Mutch D.M. (2019). EPA and DHA have divergent effects on serum triglycerides and lipogenesis, but similar effects on lipoprotein lipase activity: A randomized controlled trial. Am. J. Clin. Nutr..

[111] Rustan A.C., Christiansen E.N., Drevon C.A. (1992). Serum lipids, hepatic glycerolipid metabolism and peroxisomal fatty acid oxidation in rats fed omega-3 and omega-6 fatty acids. Biochem. J..

[112] Green C.J., Pramfalk C., Charlton C.A., Gunn P.J., Cornfield T., Pavlides M., Karpe F., Hodson L. (2020). Hepatic de novo lipogenesis is suppressed and fat oxidation is increased by omega-3 fatty acids at the expense of glucose metabolism. BMJ Open Diabetes Res. Care.

[113] Ferre P., Phan F., Foufelle F. (2021). SREBP-1c and lipogenesis in the liver: An update1. Biochem. J..

[114] Yoshikawa T., Shimano H., Yahagi N., Ide T., Amemiya-Kudo M., Matsuzaka T., Nakakuki M., Tomita S., Okazaki H., Tamura Y. (2002). Polyunsaturated fatty acids suppress sterol regulatory element-binding protein 1c promoter activity by inhibition of liver X receptor (LXR) binding to LXR response elements. J. Biol. Chem..

[115] Xu J., Nakamura M.T., Cho H.P., Clarke S.D. (1999). Sterol regulatory element binding protein-1 expression is suppressed by dietary polyunsaturated fatty acids. A mechanism for the coordinate suppression of lipogenic genes by polyunsaturated fats. J. Biol. Chem..

[116] Xu J., Teran-Garcia M., Park J.H., Nakamura M.T., Clarke S.D. (2001). Polyunsaturated fatty acids suppress hepatic sterol regulatory element-binding protein-1 expression by accelerating transcript decay. J. Biol. Chem..

[117] Song S., Attia R.R., Connaughton S., Niesen M.I., Ness G.C., Elam M.B., Hori R.T., Cook G.A., Park E.A. (2010). Peroxisome proliferator activated receptor alpha (PPARalpha) and PPAR gamma coactivator (PGC-1alpha) induce carnitine palmitoyltransferase IA (CPT-1A) via independent gene elements. Mol. Cell Endocrinol..

[118] Rudkowska I., Caron-Dorval D., Verreault M., Couture P., Deshaies Y., Barbier O., Vohl M.C. (2010). PPARalpha L162V polymorphism alters the potential of *n*-3 fatty acids to increase lipoprotein lipase activity. Mol. Nutr. Food Res..

[119] Gani O.A., Sylte I. (2008). Molecular recognition of docosahexaenoic acid by peroxisome proliferator-activated receptors and retinoid-X receptor alpha. J. Mol. Graph. Model..

[120] Berge R.K., Madsen L., Vaagenes H., Tronstad K.J., Gottlicher M., Rustan A.C. (1999). In contrast with docosahexaenoic acid, eicosapentaenoic acid and hypolipidaemic derivatives decrease hepatic synthesis and secretion of triacylglycerol by decreased diacylglycerol acyltransferase activity and stimulation of fatty acid oxidation. Biochem. J..

[121] Willumsen N., Hexeberg S., Skorve J., Lundquist M., Berge R.K. (1993). Docosahexaenoic acid shows no triglyceride-lowering effects but increases the peroxisomal fatty acid oxidation in liver of rats. J. Lipid Res..

[122] Froyland L., Madsen L., Vaagenes H., Totland G.K., Auwerx J., Kryvi H., Staels B., Berge R.K. (1997). Mitochondrion is the principal target for nutritional and pharmacological control of triglyceride metabolism. J. Lipid Res..

[123] Harris W.S., Bulchandani D. (2006). Why do omega-3 fatty acids lower serum triglycerides?. Curr. Opin. Lipidol..

[124] Li K., Huang T., Zheng J., Wu K., Li D. (2014). Effect of marine-derived *n*-3 polyunsaturated fatty acids on C-reactive protein, interleukin 6 and tumor necrosis factor alpha: A meta-analysis. PLoS ONE.

[125] Farimani A.R., Hariri M., Azimi-Nezhad M., Borji A., Zarei S., Hooshmand E. (2018). The effect of *n*-3 PUFAs on circulating adiponectin and leptin in patients with type 2 diabetes mellitus: A systematic review and meta-analysis of randomized controlled trials. Acta Diabetol..

[126] Vors C., Allaire J., Mejia S.B., Khan T.A., Sievenpiper J.L., Lamarche B. (2021). Comparing the Effects of Docosahexaenoic and Eicosapentaenoic Acids on Inflammation Markers Using Pairwise and Network Meta-Analyses of Randomized Controlled Trials. Adv. Nutr..

[127] Mozaffarian D., Wu J.H. (2011). Omega-3 fatty acids and cardiovascular disease: Effects on risk factors, molecular pathways, and clinical events. J. Am. Coll. Cardiol..

[128] Serhan C.N., Yacoubian S., Yang R. (2008). Anti-inflammatory and proresolving lipid mediators. Annu. Rev. Pathol..

[129] Serhan C.N., Chiang N., Van Dyke T.E. (2008). Resolving inflammation: Dual anti-inflammatory and pro-resolution lipid mediators. Nat. Rev. Immunol..

[130] Shearer G.C., Harris W.S., Pedersen T.L., Newman J.W. (2010). Detection of omega-3 oxylipins in human plasma and response to treatment with omega-3 acid ethyl esters. J. Lipid Res..

[131] Elajami T.K., Colas R.A., Dalli J., Chiang N., Serhan C.N., Welty F.K. (2016). Specialized proresolving lipid mediators in patients with coronary artery disease and their potential for clot remodeling. FASEB J..

[132] Polus A., Zapala B., Razny U., Gielicz A., Kiec-Wilk B., Malczewska-Malec M., Sanak M., Childs C.E., Calder P.C., Dembinska-Kiec A. (2016). Omega-3 fatty acid supplementation influences the whole blood transcriptome in women with obesity, associated with pro-resolving lipid mediator production. Biochim. Biophys. Acta.

[133] Schwarz D., Kisselev P., Chernogolov A., Schunck W.H., Roots I. (2005). Human CYP1A1 variants lead to differential eicosapentaenoic acid metabolite patterns. Biochem. Biophys. Res. Commun..

[134] Arnold C., Markovic M., Blossey K., Wallukat G., Fischer R., Dechend R., Konkel A., von Schacky C., Luft F.C., Muller D.N. (2010). Arachidonic acid-metabolizing cytochrome P450 enzymes are targets of omega-3 fatty acids. J. Biol. Chem..

[135] Oster R.T., Tishinsky J.M., Yuan Z., Robinson L.E. (2010). Docosahexaenoic acid increases cellular adiponectin mRNA and secreted adiponectin protein, as well as PPARgamma mRNA, in 3T3-L1 adipocytes. Appl. Physiol. Nutr. Metab..

[136] Tishinsky J.M., Ma D.W., Robinson L.E. (2011). Eicosapentaenoic acid and rosiglitazone increase adiponectin in an additive and PPARgamma-dependent manner in human adipocytes. Obesity.

[137] Huang F., Wei H., Luo H., Jiang S., Peng J. (2011). EPA inhibits the inhibitor of kappaBalpha (IkappaBalpha)/NF-kappaB/muscle RING finger 1 pathway in C2C12 myotubes in a PPARgamma-dependent manner. Br. J. Nutr..

[138] Carullo G., Mazzotta S., Vega-Holm M., Iglesias-Guerra F., Vega-Perez J.M., Aiello F., Brizzi A. (2021). GPR120/FFAR4 Pharmacology: Focus on Agonists in Type 2 Diabetes Mellitus Drug Discovery. J. Med. Chem..

[139] Yan Y., Jiang W., Spinetti T., Tardivel A., Castillo R., Bourquin C., Guarda G., Tian Z., Tschopp J., Zhou R. (2013). Omega-3 fatty acids prevent inflammation and metabolic disorder through inhibition of NLRP3 inflammasome activation. Immunity.

[140] Oh D.Y., Talukdar S., Bae E.J., Imamura T., Morinaga H., Fan W., Li P., Lu W.J., Watkins S.M., Olefsky J.M. (2010). GPR120 is an omega-3 fatty acid receptor mediating potent anti-inflammatory and insulin-sensitizing effects. Cell.

[141] Huang C.Y., Sheu W.H., Chiang A.N. (2015). Docosahexaenoic acid and eicosapentaenoic acid suppress adhesion molecule expression in human aortic endothelial cells via differential mechanisms. Mol. Nutr. Food Res..

[142] Mobraten K., Haug T.M., Kleiveland C.R., Lea T. (2013). Omega-3 and omega-6 PUFAs induce the same GPR120-mediated signalling events, but with different kinetics and intensity in Caco-2 cells. Lipids Health Dis..

[143] Kim J.M., Lee K.P., Park S.J., Kang S., Huang J., Lee J.M., Sato K., Chung H.Y., Okajima F., Im D.S. (2015). Omega-3 fatty acids induce Ca(2+) mobilization responses in human colon epithelial cell lines endogenously expressing FFA4. Acta Pharmacol. Sin..

[144] Watson S.J., Brown A.J., Holliday N.D. (2012). Differential signaling by splice variants of the human free fatty acid receptor GPR120. Mol. Pharmacol..

[145] Wang C.P., Lee C.C., Wu D.Y., Chen S.Y., Lee T.M. (2022). Differential effects of EPA and DHA on PPARgamma-mediated sympathetic innervation in infarcted rat hearts by GPR120-dependent and -independent mechanisms. J. Nutr. Biochem..

[146] Pirillo A., Norata G.D., Catapano A.L. (2013). LOX-1, OxLDL, and atherosclerosis. Mediators Inflamm..

[147] Duewell P., Kono H., Rayner K.J., Sirois C.M., Vladimer G., Bauernfeind F.G., Abela G.S., Franchi L., Nunez G., Schnurr M. (2010). NLRP3 inflammasomes are required for atherogenesis and activated by cholesterol crystals. Nature.

[148] Mason R.P., Sherratt S.C., Jacob R.F. (2016). Eicosapentaenoic Acid Inhibits Oxidation of ApoB-containing Lipoprotein Particles of Different Size In Vitro When Administered Alone or in Combination With Atorvastatin Active Metabolite Compared With Other Triglyceride-lowering Agents. J. Cardiovasc. Pharmacol..

[149] Mason R.P., Jacob R.F. (2015). Eicosapentaenoic acid inhibits glucose-induced membrane cholesterol crystalline domain formation through a potent antioxidant mechanism. Biochim. Biophys. Acta.

[150] Sakai C., Ishida M., Ohba H., Yamashita H., Uchida H., Yoshizumi M., Ishida T. (2017). Fish oil omega-3 polyunsaturated fatty acids attenuate oxidative stress-induced DNA damage in vascular endothelial cells. PLoS ONE.

[151] Heshmati J., Morvaridzadeh M., Maroufizadeh S., Akbari A., Yavari M., Amirinejad A., Maleki-Hajiagha A., Sepidarkish M. (2019). Omega-3 fatty acids supplementation and oxidative stress parameters: A systematic review and meta-analysis of clinical trials. Pharmacol. Res..

[152] Sherratt S.C.R., Juliano R.A., Mason R.P. (2020). Eicosapentaenoic acid (EPA) has optimal chain length and degree of unsaturation to inhibit oxidation of small dense LDL and membrane cholesterol domains as compared to related fatty acids in vitro. Biochim. Biophys. Acta Biomembr..

[153] Sherratt S.C.R., Mason R.P. (2018). Eicosapentaenoic acid inhibits oxidation of high density lipoprotein particles in a manner distinct from docosahexaenoic acid. Biochem. Biophys. Res. Commun..

[154] Wu T., Geigerman C., Lee Y.S., Wander R.C. (2002). Enrichment of LDL with EPA and DHA decreased oxidized LDL-induced apoptosis in U937 cells. Lipids.

[155] Mebarek S., Ermak N., Benzaria A., Vicca S., Dubois M., Nemoz G., Laville M., Lacour B., Vericel E., Lagarde M. (2009). Effects of increasing docosahexaenoic acid intake in human healthy volunteers on lymphocyte activation and monocyte apoptosis. Br. J. Nutr..

[156] Muralidhar B., Carpenter K.L., Muller K., Skepper J.N., Arends M.J. (2004). Potency of arachidonic acid in polyunsaturated fatty acid-induced death of human monocyte-macrophages: Implications for atherosclerosis. Prostaglandins Leukot. Essent. Fat. Acids.

[157] Novinbahador T., Nourazarian A., Asgharzadeh M., Rahbarghazi R., Avci C.B., Bagca B.G., Ozates N.P., Karbasforoush S., Khaki-Khatibi F. (2018). Docosahexaenoic acid attenuates the detrimental effect of palmitic acid on human endothelial cells by modulating genes from the atherosclerosis signaling pathway. J. Cell Biochem..

[158] Jung T.W., Park H.S., Choi G.H., Kim D., Ahn S.H., Kim D.S., Lee T., Jeong J.H. (2018). Maresin 1 attenuates pro-inflammatory reactions and ER stress in HUVECs via PPARalpha-mediated pathway. Mol. Cell Biochem..

[159] Li S., Sun Y., Liang C.P., Thorp E.B., Han S., Jehle A.W., Saraswathi V., Pridgen B., Kanter J.E., Li R. (2009). Defective phagocytosis of apoptotic cells by macrophages in atherosclerotic lesions of ob/ob mice and reversal by a fish oil diet. Circ. Res..

[160] Chang H.Y., Lee H.N., Kim W., Surh Y.J. (2015). Docosahexaenoic acid induces M2 macrophage polarization through peroxisome proliferator-activated receptor gamma activation. Life Sci..

[161] Hosseini Z., Marinello M., Decker C., Sansbury B.E., Sadhu S., Gerlach B.D., Bossardi Ramos R., Adam A.P., Spite M., Fredman G. (2021). Resolvin D1 Enhances Necroptotic Cell Clearance Through Promoting Macrophage Fatty Acid Oxidation and Oxidative Phosphorylation. Arterioscler. Thromb. Vasc. Biol..

[162] Gerlach B.D., Marinello M., Heinz J., Rymut N., Sansbury B.E., Riley C.O., Sadhu S., Hosseini Z., Kojima Y., Tang D.D. (2020). Resolvin D1 promotes the targeting and clearance of necroptotic cells. Cell Death Differ..

[163] Bouchard-Mercier A., Rudkowska I., Lemieux S., Couture P., Vohl M.C. (2013). Polymorphisms, de novo lipogenesis, and plasma triglyceride response following fish oil supplementation. J. Lipid Res..

[164] Ouellette C., Cormier H., Rudkowska I., Guenard F., Lemieux S., Couture P., Vohl M.C. (2013). Polymorphisms in genes involved in the triglyceride synthesis pathway and marine omega-3 polyunsaturated fatty acid supplementation modulate plasma triglyceride levels. J. Nutr. Nutr..

[165] Tremblay B.L., Cormier H., Rudkowska I., Lemieux S., Couture P., Vohl M.C. (2015). Association between polymorphisms in phospholipase A2 genes and the plasma triglyceride response to an *n*-3 PUFA supplementation: A clinical trial. Lipids Health Dis..

[166] Bouchard-Mercier A., Rudkowska I., Lemieux S., Couture P., Vohl M.C. (2014). Polymorphisms in genes involved in fatty acid beta-oxidation interact with dietary fat intakes to modulate the plasma TG response to a fish oil supplementation. Nutrients.

[167] Meher A., Joshi A., Joshi S. (2014). Differential regulation of hepatic transcription factors in the Wistar rat offspring born to dams fed folic acid, vitamin B12 deficient diets and supplemented with omega-3 fatty acids. PLoS ONE.

[168] Aslibekyan S., Wiener H.W., Havel P.J., Stanhope K.L., O’Brien D.M., Hopkins S.E., Absher D.M., Tiwari H.K., Boyer B.B. (2014). DNA methylation patterns are associated with *n*-3 fatty acid intake in Yup’ik people. J. Nutr..

[169] de la Rocha C., Perez-Mojica J.E., Leon S.Z., Cervantes-Paz B., Tristan-Flores F.E., Rodriguez-Rios D., Molina-Torres J., Ramirez-Chavez E., Alvarado-Caudillo Y., Carmona F.J. (2016). Associations between whole peripheral blood fatty acids and DNA methylation in humans. Sci. Rep..

[170] Hoile S.P., Clarke-Harris R., Huang R.C., Calder P.C., Mori T.A., Beilin L.J., Lillycrop K.A., Burdge G.C. (2014). Supplementation with *n*-3 long-chain polyunsaturated fatty acids or olive oil in men and women with renal disease induces differential changes in the DNA methylation of FADS2 and ELOVL5 in peripheral blood mononuclear cells. PLoS ONE.

[171] do Amaral C.L., Milagro F.I., Curi R., Martinez J.A. (2014). DNA methylation pattern in overweight women under an energy-restricted diet supplemented with fish oil. Biomed. Res. Int..

[172] Lee H.S., Barraza-Villarreal A., Biessy C., Duarte-Salles T., Sly P.D., Ramakrishnan U., Rivera J., Herceg Z., Romieu I. (2014). Dietary supplementation with polyunsaturated fatty acid during pregnancy modulates DNA methylation at IGF2/H19 imprinted genes and growth of infants. Physiol. Genom..

[173] Hoyo C., Fortner K., Murtha A.P., Schildkraut J.M., Soubry A., Demark-Wahnefried W., Jirtle R.L., Kurtzberg J., Forman M.R., Overcash F. (2012). Association of cord blood methylation fractions at imprinted insulin-like growth factor 2 (IGF2), plasma IGF2, and birth weight. Cancer Causes Control..

[174] Perkins E., Murphy S.K., Murtha A.P., Schildkraut J., Jirtle R.L., Demark-Wahnefried W., Forman M.R., Kurtzberg J., Overcash F., Huang Z. (2012). Insulin-like growth factor 2/H19 methylation at birth and risk of overweight and obesity in children. J. Pediatr..

[175] van Dijk S.J., Zhou J., Peters T.J., Buckley M., Sutcliffe B., Oytam Y., Gibson R.A., McPhee A., Yelland L.N., Makrides M. (2016). Effect of prenatal DHA supplementation on the infant epigenome: Results from a randomized controlled trial. Clin. Epigenetics.

[176] Lind M.V., Martino D., Harslof L.B., Kyjovska Z.O., Kristensen M., Lauritzen L. (2015). Genome-wide identification of mononuclear cell DNA methylation sites potentially affected by fish oil supplementation in young infants: A pilot study. Prostaglandins Leukot. Essent. Fat. Acids.

[177] Sampath H., Ntambi J.M. (2004). Polyunsaturated fatty acid regulation of gene expression. Nutr. Rev..

[178] Harris W.S., Miller M., Tighe A.P., Davidson M.H., Schaefer E.J. (2008). Omega-3 fatty acids and coronary heart disease risk: Clinical and mechanistic perspectives. Atherosclerosis.

[179] Rundblad A., Larsen S.V., Myhrstad M.C., Ottestad I., Thoresen M., Holven K.B., Ulven S.M. (2019). Differences in peripheral blood mononuclear cell gene expression and triglyceride composition in lipoprotein subclasses in plasma triglyceride responders and non-responders to omega-3 supplementation. Genes. Nutr..

